# Intrathecal pemetrexed for leptomeningeal metastases in a patient with *ALK*-rearranged lung adenocarcinoma: a case report

**DOI:** 10.1007/s00280-024-04735-8

**Published:** 2024-12-17

**Authors:** Emelie Gezelius, Maria Planck, Bassam Hazem, Seema Nagpal, Heather Wakelee

**Affiliations:** 1 Division of Oncology, Department of Clinical Sciences Lund, Lund University, Barngatan 4, Lund, SE-221 85 Sweden; 2 Department of Respiratory Medicine and Allergology, Skåne University Hospital, Lund, Sweden; 3 Department of Neurology, Stanford University School of Medicine, Stanford Cancer Institute, Stanford, USA; 4 Division of Oncology, Department of Medicine, Stanford University School of Medicine, Stanford Cancer Institute, Stanford, USA

**Keywords:** Intrathecal pemetrexed, Leptomeningeal metastasis, Non-small cell lung cancer, ALK, Case report

## Abstract

Progressive leptomeningeal metastases (LM) are associated with intractable neurological symptoms and a poor prognosis, and effective treatment options are limited. Intrathecal (IT) pemetrexed has been shown to confer clinical benefit in lung adenocarcinoma, yet our understanding of the efficacy and safety of the treatment is limited. We report a patient with a long-standing history of leptomeningeal disease due to *ALK*-positive adenocarcinoma of the lung, previously controlled by increased doses of lorlatinib (125 mg/day). Rapid LM progression prompted the start of IT pemetrexed, after which the patient experienced immediate clinical improvement. The case provides additional support that IT pemetrexed can offer symptomatic relief and may be considered as a treatment option in advanced LM. Furthermore, the case illustrates that an increased dose of lorlatinib may efficiently control LM in patients with *ALK*-rearranged NSCLC, following progression on standard lorlatinib dosage.

## Introduction

Leptomeningeal metastases (LM) are associated with intractable neurological symptoms and a poor prognosis [1, 2]. LM is more frequently seen in non-small cell lung cancer (NSCLC) patients harboring oncogenic driver alterations [3–5], where several of the molecularly targeted agents have proved to efficiently reduce intracranial manifestations. Still, treatment-resistance inevitably develops, and the management of LM then poses a therapeutic challenge. Intrathecal (IT) pemetrexed has been demonstrated to induce clinical and radiologic responses and may be considered as a treatment option in patients with progressive LM secondary to lung adenocarcinoma [6, 7]. However, our understanding of the efficacy and safety of intrathecally administered pemetrexed is still limited.

Here, we present a patient with a long-standing history of LM due to anaplastic lymphoma kinase (*ALK*) -rearranged NSCLC, where IT pemetrexed rapidly improved neurological symptoms and quality of life. Our case illustrates several considerations that are made for long-term surviving patients with *ALK*-positive lung cancer during their late lines of treatment and provides additional support that IT therapy is worth evaluating in LM trials that include both oncogene-driven and non-oncogene driven lung cancers.

## Case

**Fig. 1:**
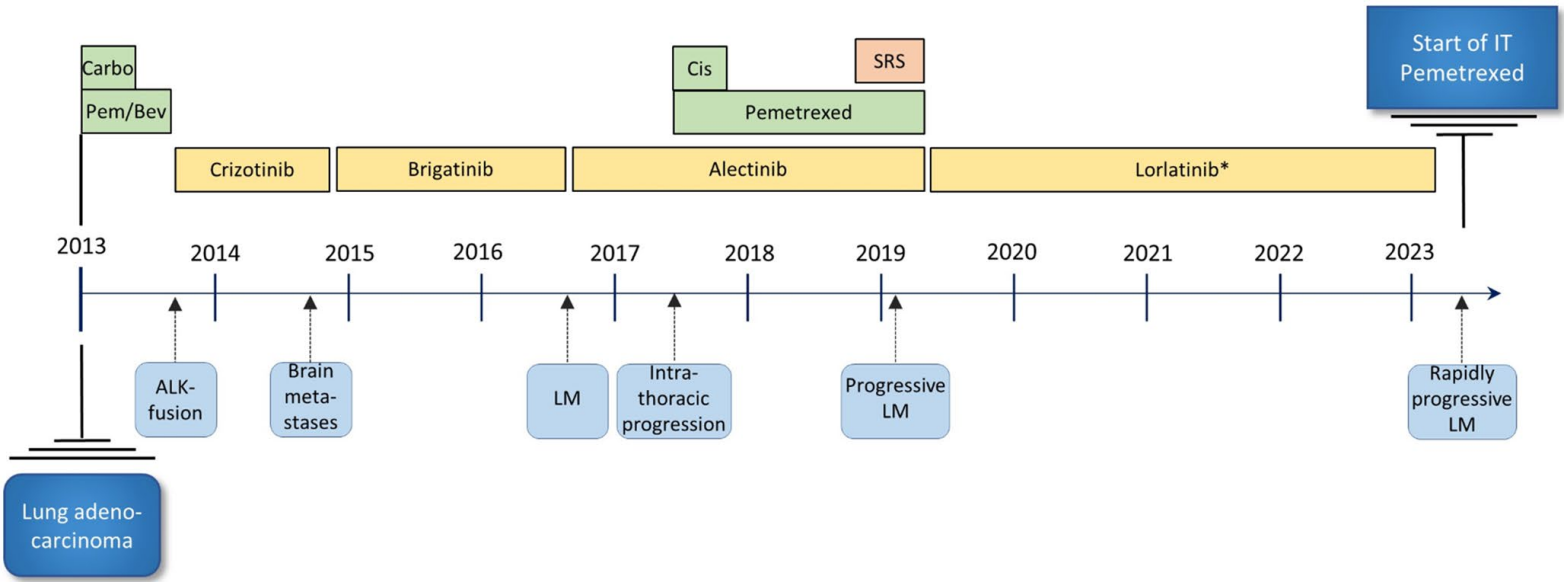
Summary of patient history. Leptomeningeal metastases were cytologically confirmed in 2016. Both alectinib and lorlatinib induced durable responses of the leptomeningeal disease. Note, brigatinib was accessed through a clinical trial. Carbo = Carboplatin; Pem/Bev = Pemetrexed/Bevacizumab; Cis = Cisplatin; SRS = Stereotactic radiosurgery towards three cerebral metastases; IT = Intrathecal; ALK = Anaplastic lymphoma kinase; LM = Leptomeningeal metastases. *Increased dose of lorlatinib (125 mg/day) from October 2020

**Fig. 2:**
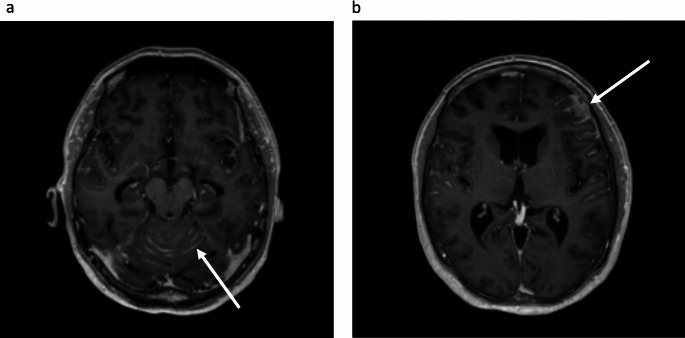
Magnetic resonance imaging of the brain. The images show extensive, abnormal contrast enhancement. The white arrows indicate meningeal contrast enhancement in the cerebellum (**a**) and left frontal sulci (**b**)

A 47-year-old woman with a 10-year history of metastatic *ALK*-positive NSCLC presented in September 2023 with increasing headache, nausea, visual disturbances and tiredness. She had previously received multiple lines of treatment, including chemotherapy and *ALK*-targeting tyrosine kinase inhibitors, as summarized in Fig. 1. LM was diagnosed in 2016 and had efficiently been controlled initially by alectinib, then by lorlatinib, and most recently by the increased dose of lorlatinib 125 mg once daily, for the last three years. In September 2023 magnetic resonance imaging (MRI) of the brain showed extensive progression of LM (Fig. 2), extending along several cranial nerves including the optic, oculomotor, and acoustic nerves.

An attempt was made to re-challenge with brigatinib, but this was rapidly discontinued due to the development of bradycardia. The patient suffered from severe neurological symptoms, resistant to high doses of corticosteroids. Consequently, she was unable to sit upright and had an Eastern Cooperative Oncology Group (ECOG) performance status of 4. Ophthalmologic assessment revealed near complete loss of vision on the right eye and a visual acuity of 0.03 on the left eye, according to the decimal chart. The decision was made to start intrathecal pemetrexed. The patient received folic acid 1 mg/day and an intramuscular injection of vitamin B12 (1 mg). A lumbar puncture was performed using sterile technique according to hospital standards. Six mL of cerebrospinal fluid (CSF) were withdrawn and sent for cytology and molecular analysis. Malignant cells were verified and, by targeted sequencing (Oncomine™ Lung cfDNA Assay, Ion S5™, ThermoFisher) of cell-free DNA (cfDNA) from the CSF, we could detect the presence of the *ALK* resistance mutation L1196M, with an allele frequency of 25.5%. This compares to an identification of the same mutation, but below the technically approved limit of detection, in the corresponding analysis 8 months earlier. In contrast, no tumor-derived cfDNA was detected in plasma at either of the two sample occasions. Furthermore, centrifugation of CSF resulted in a cell pellet from which RNA was extracted (Qiagen AllPrep DNA/RNA Mini Kit) and subjected to next generation sequencing (Illumina TruSight Oncology 500). This enabled the identification of the previously unknown *ALK* fusion EML4-ALK_E13:A20.

Dose 1 of IT pemetrexed was administered as 30 mg of pemetrexed (Hospira/Pfizer) diluted in 0.9% normal saline for a total volume of 5 mL, followed by prednisolone 25 mg (Precortalone Aqueous, ACE Pharmaceuticals, 1 mL). One day later, the headache and nausea had improved drastically. The patient was able to sit upright, and appetite had returned. As performance status continued to normalize, the second dose was increased to 50 mg pemetrexed, administered on day 6. Subsequent cycles of IT pemetrexed and prednisolone were administered via lumbar puncture, with 3-week intervals.

After 3 injections, the patient was able to walk short distances without support. She had regained visual acuity of 1.0 (decimal acuity) on the left eye, and an MRI scan showed stable disease. Computed tomography of the chest and abdomen revealed no signs of extracranial disease progression. Two months following the initial clinical improvement, the patient experienced a return of the headache and nausea. These symptoms were reduced by shortening the treatment interval to 2.5 weeks. However, a gradual deterioration was noted, with fatigue, visual loss and transient episodes of hearing loss. Mild radiological progression was seen on a repeated MRI scan. As the IT therapy appeared to alleviate the headache and nausea, further cycles were planned. The patient received 6 injections in total, before treatment was discontinued due to enrollment into a clinical trial. Three months later the condition deteriorated, and the patient was transitioned to palliative care only. She passed away eleven years after the initial diagnosis of metastatic NSCLC.

## Discussion

Treatment options are limited in progressive leptomeningeal disease. As illustrated by the present case, IT pemetrexed may induce rapid clinical improvement. This is consistent with two prospective studies, reporting high clinical response rates of 84.6% [6] and 82.6% [7], respectively. It is difficult to determine to what extent the IT corticosteroids or the CSF pressure relief caused by the lumbar puncture may have contributed to the positive effects in our patient. Nonetheless, the intervention clearly improved the patient’s quality of life for several months.

No consensus has yet been reached regarding the optimal treatment regimen of IT pemetrexed, as existing literature displays great variations in dosing and frequency of administrations [6–8]. In a retrospective study, pemetrexed was injected in incrementally escalating doses twice weekly in the first month, once per week during the second month, followed by monthly injections in the subsequent months [8]. The phase 1/2 study by Fan et al. established 50 mg as the recommended dose administered twice during the first week followed by 3-week intervals for the first month, and subsequently, once every 4 weeks [6]. Considering the palliative situation, longer treatment intervals have the advantage of fewer hospital visits, and our aim was therefore to follow this suggested regimen. However, during the disease course that followed, we shortened the treatment interval to limit symptomatic progression between doses.

IT pemetrexed appears generally well tolerated. The main side-effects previously reported include myelosuppression and neurological toxicity, involving headache, nausea, and focal neurological deficits [6, 7, 9]. Thus, it may be challenging to discriminate between neurological toxicity and symptoms caused by the leptomeningeal disease itself. With the exception of temporary fatigue, our patient did not develop any obvious side-effects of the treatment.

The intracranial efficacy of lorlatinib and other 2nd and 3rd generation TKIs is well documented [10–12]. The present case demonstrates survival extending beyond 9 years from the first finding of CNS metastases. It is worth noting that after radiological LM progression during standard lorlatinib dosage (100 mg/day), an increased dose (125 mg/day) efficiently controlled the LM for several years, with limited toxicity. To our knowledge, this has not previously been described.

Whereas no circulating cfDNA could be detected in plasma from our patient, sequencing of cfDNA in CSF identified the resistance mutation *ALK* L1196M with increasing allele frequency following the course of the disease. This confirms the clinical significance of CSF-based cfDNA analysis in patients with progressive LM. In a previous retrospective analysis of paired CSF and plasma samples from 11 patients with *ALK*-rearranged NSCLC, the CSF-based approach detected both targetable alterations and resistance mechanisms with a higher sensitivity compared to the plasma testing [5].

## Conclusion

Intrathecal pemetrexed may offer symptomatic relief and clinical improvement in patients with progressive LM due to lung adenocarcinoma, where no targeted treatment options remain. Further studies are needed to confirm the efficacy of IT pemetrexed, to determine the optimal dosing and timing of the therapy, and to establish a role for CSF-based molecular analyses in detection of resistance mechanisms, response evaluation, and disease monitoring.

## Data Availability

No datasets were generated or analysed during the current study.
